# Mind Your Step: the Effects of Mobile Phone Use on Gaze Behavior in Stair Climbing

**DOI:** 10.1007/s41347-017-0022-6

**Published:** 2017-11-28

**Authors:** Flora Ioannidou, Frouke Hermens, Timothy L. Hodgson

**Affiliations:** School of Psychology, University of Lincoln, Brayford Pool, LN6 7TS Lincoln, UK

**Keywords:** Stair walking, Visual attention, Mobile phone use, Distraction, Eye tracking

## Abstract

Stair walking is a hazardous activity and a common cause of fatal and non-fatal falls. Previous studies have assessed the role of eye movements in stair walking by asking people to repeatedly go up and down stairs in quiet and controlled conditions, while the role of peripheral vision was examined by giving participants specific fixation instructions or working memory tasks. We here extend this research to stair walking in a natural environment with other people present on the stairs and a now common secondary task: using one’s mobile phone. Results show that using the mobile phone strongly draws one’s attention away from the stairs, but that the distribution of gaze locations away from the phone is little influenced by using one’s phone. Phone use also increased the time needed to walk the stairs, but handrail use remained low. These results indicate that limited foveal vision suffices for adequate stair walking in normal environments, but that mobile phone use has a strong influence on attention, which may pose problems when unexpected obstacles are encountered.

## Introduction

Stair walking is a common, but surprisingly hazardous activity. In the UK, more than 1000 people die every year from falls from stairs (Hill et al. 2000), and more than 300,000 are treated for injuries following a fall from stairs (HASS and LASS Home & Leisure Accident Surveillance System 2002). Older people are particularly at risk due to age-related impairments of vision, strength and balance. Despite these risks, around one third of interviewed elderly indicated to leave household objects on stairs, and one in three continued carrying difficult objects on stairs (Hill et al. 2000). Given these numbers, it is vital to understand the cognitive processes involved in stair walking to determine how to best prevent falls.

Studies in which the visibility of stairs was modulated during normal walking have suggested that having a good view of the stairs is crucial to the task (Adolph and Eppler 1998; Gibson 1958). For example, placement of the foot (Timmis et al. 2009) as well as walking speed (Marigold and Patla 2008) has been found to be affected by blocking the view of the lower visual field (as in when carrying a large box). Likewise, reduced contrast while walking in a virtual reality environment reduces walking speed and increases the number of contacts with obstacles (Hassan et al. 2007). The use of multifocal glasses has been found to increase the risk of falling (Johnson et al. 2007; Lord et al. 2002). Visual impairments are more common in the elderly, and this could be a factor explaining the higher incidence of accidents during falls in this age group (Startzell et al. 2000). These results agree with findings in other walking activities, such as stepping on targets and walking around obstacles. For example, studies in which monocular vision (Hayhoe et al. 2009), blurred vision (Buckley et al. 2005) and absent vision (Buckley et al. 2008) were investigated demonstrate the importance of adequate vision when walking around obstacles.

While detailed visual information processing relies on foveating the objects of interest, the visual system can also extract relevant information from the scene using information outside the fovea. Studies on stair walking with and without distractor tasks have suggested that while people often fixate the stairs without distraction (around 50% of the time), they can walk stairs safely when given specific fixation or secondary tasks, with very few direct fixations on the stairs (Miyasike-daSilva et al. 2011; Miyasike-daSilva and McIlroy 2012). Higher percentages (75 to 90% of fixations) were found by Zietz and Hollands (2009), but these numbers were specific for the middle section of the stairs. Similarly, based on observing stair walkers, Rosenbaum (2009) found that people most often looked down twice during an eight steps descent, with some walkers never looking down. Reduced numbers of stair fixations, however, were not found to increase handrail use or imbalances (Miyasike-daSilva and McIlroy 2012), although variability across participants in such factors increased with distraction (Miyasike-daSilva and McIlroy 2016). The sufficiency of extrafoveal information alone is supported by findings showing that during obstacle avoidance, obstacles were rarely fixated (Franchak and Adolph 2010). Reliance on extrafoveal vision, however, may depend on the scene. In the presence of moving obstacles, such as other people, foveation of potential colliders is frequent (Jovancevic et al. 2006), particularly when other pedestrians’ walking behavior is unpredictable (Jovancevic-Misic et al. 2007; Jovancevic-Misic and Hayhoe 2009). This suggests that while foveal information extraction may be the default option, it is not strictly necessary, meaning that people can do with a brief glance of the stairs and information gained from peripheral vision. Such results, however, may depend on whether stair walking is studied in a quiet environment, and whether other people are present. Taking studies of gaze behavior from the lab into the real world may provide further information on how day-to-day stair navigation is performed.

Past studies have relied on highly controlled stair walking conditions and specific gaze instructions or visual tasks to influence the time spent looking at stairs to determine the role of extrafoveal vision in stair navigation (Miyasike-daSilva and McIlroy 2012, 2016). For example, participants were instructed to walk a 7-step staircase located in a quiet laboratory without a secondary task, while fixating a target at the end of the stairs, performing a visual reaction time task, or an auditory reaction time task (Miyasike-daSilva and McIlroy 2012). Such instructions and conditions, while highly controlled, are unlike secondary tasks that people tend to perform while during natural stair walking. With recent developments in mobile phone technology, it has become much more common to use hand-held devices during locomotion. While with older phones, the most common activity was talking on the phone, leaving visual input intact, modern phones increasingly involve visual engagement with the device for looking up information, sending texts, using maps or playing games, meaning that more and more people’s visual attention may be distracted away from their walking. The decremental effects of mobile phone use on people’s (visual) attention has been well documented, both during locomotion and driving. For example, a sixfold increase in pedestrian injuries related to mobile phone use was found during the rise of smart phone technology (between 2004 and 2010, Nasar and Troyer 2013). Talking on the phone has been found to reduce memory for objects planted along the route and to increase unsafe crossing at crosswalks (Nasar et al. 2008). Phone users were found to walk more slowly, to change direction more often and notice unusual activities less often (Hyman et al. 2010). During virtual reality road crossing, phone use distracted attention away from traffic, led to unsafe crossing behavior and more collisions and close calls with upcoming traffic (Stavrinos et al. 2009), while other work found similar risks from texting and listening to music while walking (Schwebel et al. 2012). In driving, the dangerous effects of concurrent phone use have been known for longer, possibly because of direct danger to others. Driving behavior has been shown to be affected by mobile phone use (Engström et al. 2005; Törnros and Bolling 2005), as is the distribution of overt attention (Konstantopoulos et al. 2010), recognition memory (Strayer et al. 2003), and reaction times to slowing of the car ahead (Lamble et al. 1999), all of which can increase the risk of car related accidents both during and after phone use (Redelmeier and Tibshirani 1997). A recent high profile case showed the danger of walking while using a mobile phone, when two men fell down a cliff while playing a game on their phones (CNN 2016). Only one study appears to have specifically addressed the influence of phone use on stair walking (Lester et al. 2016), but focused on only the first two steps of the walk. Results suggested a reduction of fixations on the stairs during phone use, in particular for the second step. Based on these results, and those in stair locomotion with highly controlled distractor tasks (Miyasike-daSilva and McIlroy 2012), and the effects of phone use on locomotion and driving (Hyman et al. 2010; Konstantopoulos et al. 2010), we expect phones to strongly attract attention away from the stairs, and to reduce walking speed, but it is unclear how phone use affects fixations away from the phone and measures of instability, such as hand rail use.

With this information in mind, the aim of the present study was to extend previous studies on gaze behavior during stair walking in controlled environments (Miyasike-daSilva et al. 2011; Lester et al. 2016; Zietz and Hollands 2009) and controlled task instructions (Miyasike-daSilva and McIlroy 2012, 2016; Zietz and Hollands 2009) to the real world. Participants were asked to walk three sets of stairs in normal day-to-day conditions (with other people present) with or without using a mobile phone. Using a mobile eye tracker, gaze behavior was recorded, while participants’ use of the handrail was monitored using video recordings of participants navigating the stairs. As no security measures could be put in place in case of falls, the study was limited to walking up the stairs (as epidemiological studies have shown that falls from stairs occur more often when walking down the stairs, Svanström (1974)). To examine whether gaze behavior, activities on stairs and rail use depend on gender or age, we also asked participants about their past experience of falls and whether they often used the phone or consumed food while on the stairs.

## Methods

### Participants

Forty participants (23 females and 17 males) took part in the study, recruited using posters, an online research participation system and by word of mouth. Participants were a mixture of undergraduate and postgraduate students, university staff and members of the general public, and were between 19 and 68 years of age. Their vision was either corrected with contact lenses, or participants had normal vision without correction, as the eye tracker used did not allow for correction with glasses. Participants recruited via the poster received *£*5 for their time, those recruited via the online system received course credits, and those taking part during a public engagement event participated to learn about the latest eye tracking technology without financial reimbursement. All participants provided written consent for their participation in the study that was approved by the School of Psychology, University of Lincoln, UK, ethics committee.

### Apparatus

**Fig. 1:**
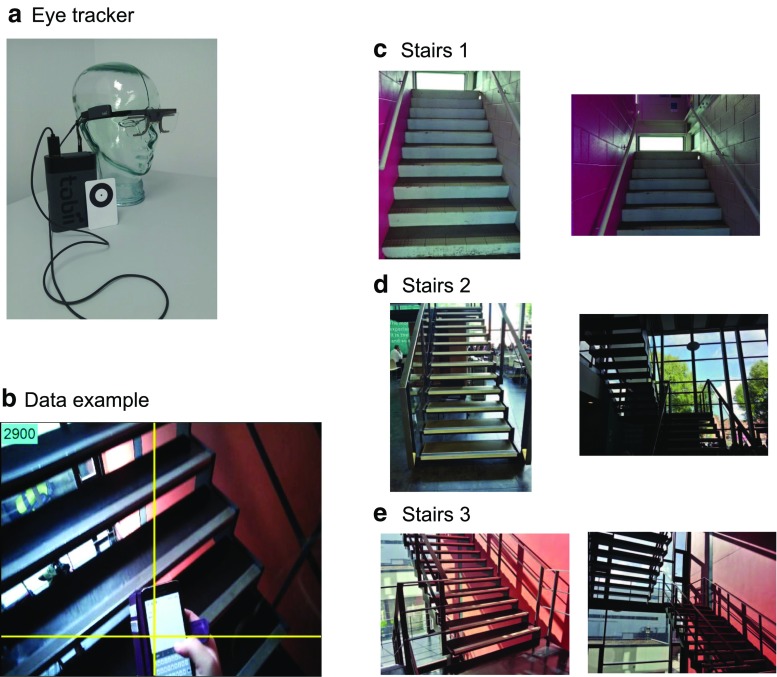
**a** Photograph of the eye tracker used (Tobii 2 glasses), showing the glasses, the recording unit and the calibration target. **b** Example of a data frame used for coding. The crosshair in the image indicates the point of view of the participant. The ROI coded for this frame would be “phone.” **c**, **d**, **e** Photographs of the three staircases that participants were asked to climb. The first stairs was located in a building adjacent to a footbridge and had solid walls and steps. The second stairs led from the ground floor of the main building of the university of Lincoln to the first floor and was surrounded by glass panels and had see-through steps. The third stairs led from the first floor to the second floor of the same building and had similar properties

Eye movements were recorded with a Tobii 2 glasses eye tracker (see Fig. 1a for an image). The Tobii 2 glasses system consists of a recording unit and head-gear in the form of a pair of glasses. The head-gear was secured by means of a strap at the back of the participants’ heads, and the recording unit was typically carried inside a pocket or attached to a belt (a back-pack was offered to participants having neither option, so that their hands were free to use for holding the handrail or the mobile phone). The Tobii 2 glasses system records a head-centered video image from the participants’ point of view (see Fig. 1b for an example) at a frame rate of 25Hz. Gaze position is recorded at 50 Hz, but since the corresponding video was sampled at half that rate, these data were down-sampled to 25 Hz to be aligned with the video data. Gaze position was derived from measurements from both eyes (binocular eye tracking), pupil and corneal reflection signals, combined into a 3D gaze coordinates on the basis of 3D models of both eyes (for our analyses, we only used the horizontal and vertical coordinates). The direction of the head-centered camera inside the system is fixed, resulting in a field of view of 52 degrees vertically and 82 degrees horizontally. Calibration of the system involves participants fixating a calibration target placed at a distance of around 1 to 1.5 m. While walking up one of the three stairs, participants used a mobile phone to type in a text message. This could be either the participants’ own phone (often a smart phone, but not for all participants), or the phone provided by the experimenter (an old style phone without a touch screen).

### Stimuli

Each participant climbed three different staircases located on the University of Lincoln campus (see Fig 1c–e, for photographs). The first staircase was located in a small building attached to the footbridge on campus. It was directly surrounded by concrete walls and had steps made of concrete. The second staircase was located inside the main building of the university, connecting the ground floor to the first floor, and had steps that were separated by spaces, so that the area below could be seen through the steps. The walls surrounding this staircase were made of glass, so that people could see the remainder of the building while climbing the stairs. The third staircase was also located in the main building and also had space between the steps, but was surrounded by solid walls on the left and right, and windows to the outside of the building ahead. All three staircases consisted of two sections, separated by a horizontal platform. They all had metal handrails on both sides of the stairs. The dimensions of the various staircases are specified in Table 1.

**Table 1:** Properties of the three staircases used in the study, providing the width of the stairs, the step height, the number of steps, and the vertical distance from the step to the handrail

Staircase	Step width (cm)	Step height (cm)	Number of steps	Step to handrail (cm)
First	116	18	28	89
Second	115	18	25	98
Third	162	18	26	92

### Design

Participants climbed the three staircases in an order controlled by a Latin square, so that the ordinal position of each stair in the sequence was counter-balanced across participants. Orthogonally to this order manipulation, the use of a cell phone while walking one of these stairs was counterbalanced across participants (so that for each staircase, around one third of participants used a mobile phone, but no participant used a phone on more than one set of stairs).

### Procedure

Participants all provided written consent before taking part in the study. They were fitted with the Tobii 2 glasses and the calibration procedure was performed (involving the fixation of a single fixation target at about an arm’s length). Two stairs were climbed without specific instruction, whereas for a third set of stairs, the experimenter informed participants that they had to try and construct a text message on their phone while climbing the stairs with information about themselves (e.g., “Hello my name is (name), i am (age), i am a (gender), and i am working at/i am a student”). Participants were guided from one staircase to another by the experimenter, who also made sure that participants were safe while climbing the stairs. Participants only ascended staircases to avoid falls, and lifts were used to move participants down floors. While participants climbed the stairs, the experimenter filmed the participant using the in-built camera of a mobile phone (1080p video image at 30fps), so that the handrail and mobile phone use could be analyzed offline and any other unexpected events were documented. After the stair climbing tasks, participants completed a short questionnaire about their experience walking stairs.

### Data Analysis

Data were preprocessed to extract the horizontal and vertical gaze location for each sample from the raw eye movement recordings, using a custom-built Perl script, after which gaze locations were combined with the head-centered video images using a Matlab script. Using another custom-built script in the same programming language, gaze locations were manually classified on a frame by frame basis according to what area they were directed to, with the following categories: one step ahead, two steps ahead, three steps ahead, four or more steps ahead, the handrail, the phone, the wall, the floor, people, “other”, and “unclear” category (e.g., during blinks or when participants looked down, and the gaze position fell outside the video image).

To examine to what extent the coding depended on the coder, approximately 25% of the data were independently coded by a second coder, yielding a 75.5% agreement, and a Cohen’s *κ* of 0.704, considered to be ‘substantial’ (McHugh 2012). Coders disagreed most often on which step participants were fixating (e.g., 1 step ahead versus 2 steps ahead; coder 2 counted the steps on the basis of the visible steps alone, whereas coder 1 also counted steps not visible in the image; a total of 12.8% of observations), whether participants were fixating a step or the floor (the final step was part of the floor section between the stairs; 0.73% of observations), whether they were fixating the wall or ‘other’ (e.g., posters on the wall; 2.92% of observations), and whether participants fixated the phone or “unclear” (coder 1 assumed missing values preceded by a fixation on the phone to be on the phone, whereas coder 2 coded these as “unclear”; 4.48% of observations). For the remainder of the data analysis, the coding of the first coder was used (who coded all data). For the interpretation of the data, the disagreement particularly for the specific step fixated needs to be taken into consideration.

For the computation of average eye movement data, such as the dwell times on the various areas of interest, the data from the individual samples were used. For example, to compute the dwell times on the phone, the total number of samples on the phone was divided by the total number of samples for that participant. This method avoids the need for fixation detection, which can be difficult in mobile eye tracking data during locomotion, where participants make a combination of saccades, smooth pursuit eye movements, and vestibular ocular reflex (VOR) eye movements, and where fixation on an object can be affected by head and body movements.

## Results

### Gaze Behavior

**Fig. 2:**
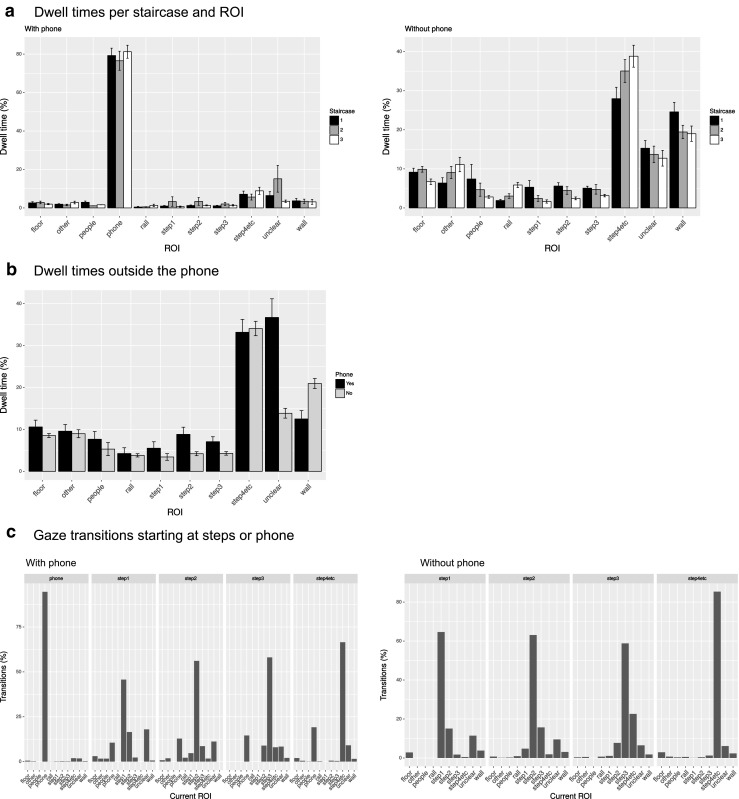
**a** Dwell times plotted separately for the different stairs when people were using their phone (left) and were not using their phone (right). Note that comparisons between conditions in the “with phone” condition are always between participants, while those in the “without phone condition” are a mixture of between and within comparisons, due to the nature of the design of the study. **b** Dwell times as a percentage of the overall time not spent looking at the phone, comparing with phone and without phone conditions, pooled across the three stairs. **c** Frequencies of ROIs fixated after fixating a particular ROI (shown in panels). The error bars in the data plots show the standard error across the means (between subject standard errors)

Figure 2a plots the time people spent looking at the various regions of interest as a percentage of the total time spent on each stair, showing on the left data for when people were using the phone, and on the right the data without use of the phone. The plots suggest that people’s overt visual attention was shifted strongly to the phone when using the phone, with little difference between the different stairs walked. Because of the small differences between stairs, and because the design did not allow for a full factorial ANOVA, dwell times were compared across stairs for people using a phone and people not using a phone, using Welch’s two samples *t* tests for each regions of interest (ROIs). Besides a significant difference for the ROI, “phone” (*t*(23) = 5.19, *p* < 0.001), significant differences were found for “floor” (*t*(62.6) = 9.87, *p* < 0.001), “other” (*t*(42.8) = 6.11, *p* < 0.001), “rail” (*t*(37.7) = 5.23, *p* < 0.001), three steps ahead (*t*(61.6) = 5.80, *p* < 0.001), four steps ahead (*t*(55.0) = 11.9, *p* < 0.001), and “wall” (*t*(55.1) = 11.8, *p* < 0.001).

The question arises whether, when not looking at their phone, people distribute their attention similarly to walkers without a phone. Figure 2b examines whether people with a phone tend to look at the same regions of interest when not looking at their phone (discarding this the spent looking at the phone) as people not using their phone. Independent sample *t* tests of dwell times on ROIs across the stairs showed significantly more time spent looking at the wall (*t*(43.2) = 3.53, *p* < 0.001) without a phone and significantly more “unclear” gazing with a phone (*t*(44.4) = 4.97, *p* < 0.001), which could be fixations on the phone that fell outside the recording window.

Figure 2c examines where participants look after they either looked at the stairs or the phone. For each of these areas (shown in panels), it shows which region of interest (ROI) is fixated next. These plots show that participants continue to fixate the same area often, but it also shows that participants progressively look further away from the current step (e.g., shift gaze from one step ahead to two steps ahead, or from two steps ahead to three steps ahead). When looking far ahead on the stairs (4+ steps ahead), they often look somewhere else (the floor, wall, or phone). When looking away from the phone, they often look further ahead on the stairs, rather than close to where they are stepping.

**Fig. 3:**
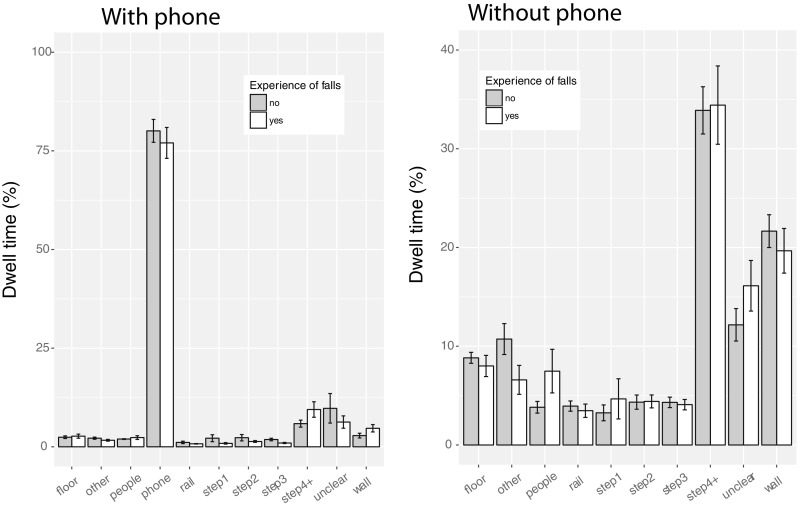
Comparison of dwell times for people with and without experience of past falls on stairs, when using a phone (left) or not using a phone (right). The error bars in the data plots reflect the standard error of the mean across participants (between subject standard errors)

In our questionnaire (see later in the results section), 14 of our 40 participants reported having experienced a fall on stairs sometime in the past. To examine whether such experience influences gaze behavior on stairs, Fig. 3 plots participants’ dwell times on the different regions of interest separately for people with past falls and those without. No effects of such fall experience were found. For stair walking without a phone, there is a non-significant tendency to look more at people for a past fall (*t*(5.62) = 1.05, *p*= 0.34). For walking with a phone, there are no obvious differences between the two groups.

### Walking Time

**Fig. 4:**
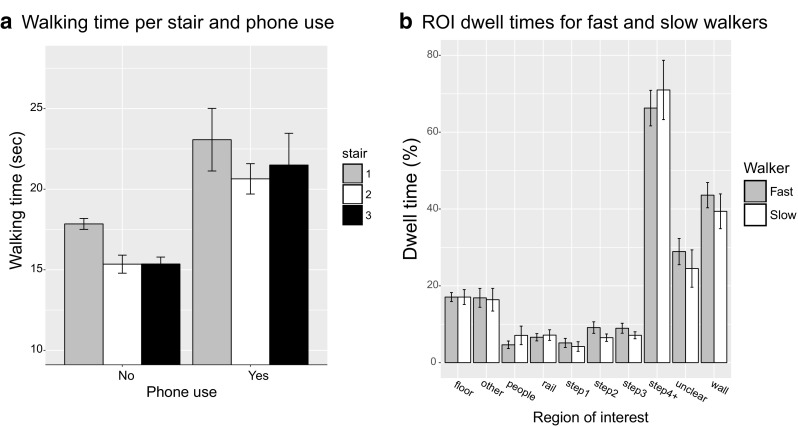
**a** Time needed to walk each of the stairs, with or without a phone. **b** Dwell times for the different ROIs for slow and fast walkers. Error bars show the standard of the mean across participants

Figure 4a shows how long it took participants to walk each of the stairs with and without a phone. Independent sample *t* tests showed a marginally significant effect of phone use for the stair 1 (*t*(13.8) = 2.65, *p* = 0.019, *d* = 1.17), and significant increases in walking times when using a phone for stairs 2 (*t*(22.4) = 4.84, *p* < 0.001, *d* = 1.71) and stairs 3 (*t*(12.1) = 3.04, *p* = 0.010, *d*= 1.48). Figure 4b examines whether the distribution of attention is different for people walking up the stairs quickly or more slowly (median split, no phone stairs only, average across stairs). Independent sample *t* tests revealed no significant differences in dwell times to the various regions of interest for slow and fast walkers (smallest *p* value = 0.15).

### Rail Use

**Fig. 5:**
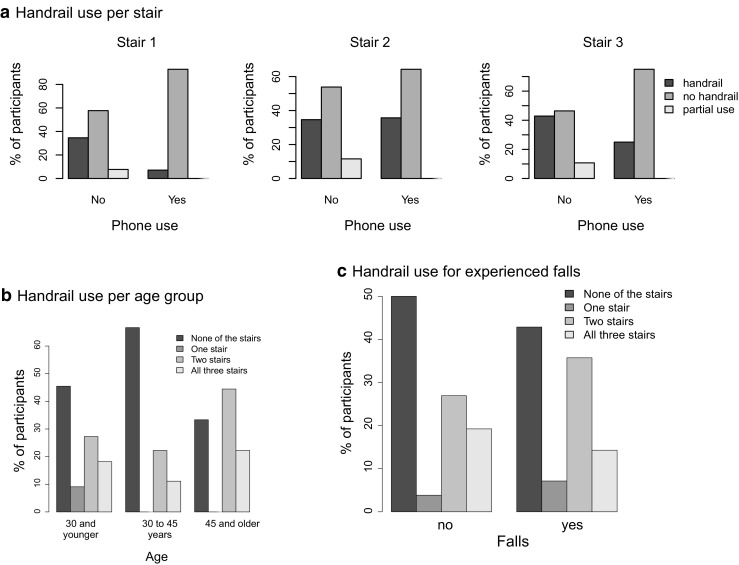
Percentage of people using the handrail all of the stairs, part of the stairs, or not using the handrail, for the three different stairs and with and without phone use. **a** Handrail use per stair. **b** Handrail use per age group. **c** Handrail use for experienced falls

Figure 5a shows the percentage of people using the handrail across the different conditions. Proportion tests showed that only in the no phone condition of stair 1, more people chose not to use the rail than to (partially) use the rail (*χ* ^2^(1) = 8.64, *p*= 0.0033). Fisher exact tests revealed a marginally significant effect of phone use on the use of the handrail for stair 1 (*p*= 0.060), but no significant effects for stair 2 (*p*= 0.67) and stair 3 (*p*= 0.23). Figure 5b examines whether rail use depends on age. While there is a trend towards more rail use in the older age group, this effect does not reach statistical significance in a Fisher exact test (*p*= 0.81). The same holds for past experience with falls, which did not lead to increased use of the handrail (Fig. 5c; *p*= 0.91).

### Questionnaire

**Fig. 6:**
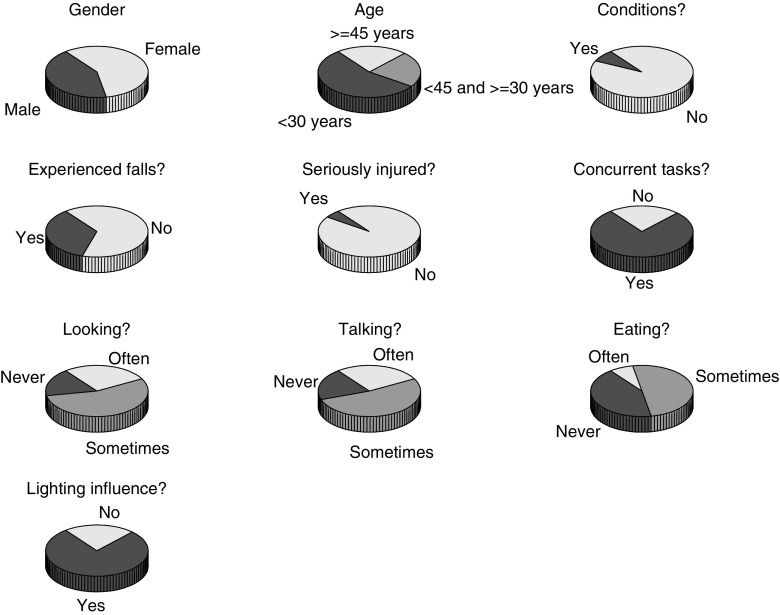
Results of the questionnaire, asking the participants gender (top left), age (top center), whether they had conditions that influenced their walking (top right), whether they had ever experienced a fall from stairs (second row left), whether they were seriously injured in a fall from stairs (second row middle), whether they ever performed other tasks while walking the stairs (second row right), whether these other tasks involved looking at their phone (third row left), talking on the phone (third row middle), or eating (third row right), and whether they thought poor lighting conditions affected walking on stairs (bottom row left)

Figure 6 provides an overview of the questionnaire results. Not many participants reported conditions that affected walking or having been seriously injured from falls on stairs, but a fairly large proportion of participants reported having had at least one fall from stairs (not significantly different from 50%, *p*= 0.082), in agreement with the large number of people in the general population presenting to healthcare providers with injuries from such falls. Around three quarters of our participants reported being engaged in other tasks when walking the stairs, which involved looking at phones, talking on phones and eating. Finally, around three quarters thought lighting conditions influenced walking on stairs.

**Fig. 7:**
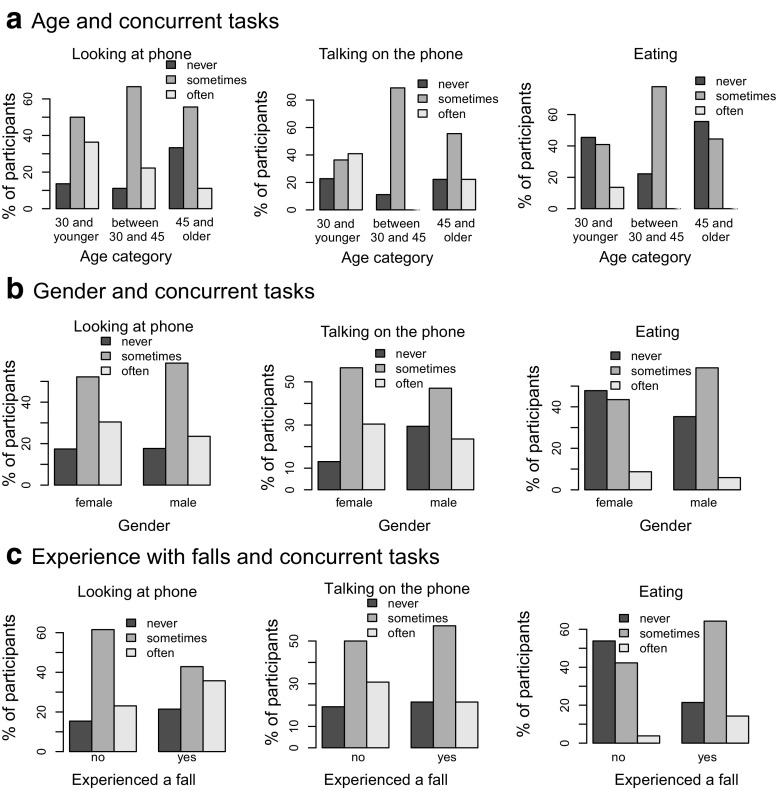
Participants reporting looking at and speaking on the phone or eating while walking the stairs, shown separately for **a** young, middle age and older participants, **b** male and female participants, **c** participants who reported having experienced a fall on stairs

Figure 7 examines whether age, gender or past falls influence whether participants report using the phone or eating while walking stairs. While younger participants appear to more often look at their phone while walking the stairs, a Fisher exact test did not reveal a significant difference with the other age groups (*p*= 0.55). The same holds for talking on the phone (*p*= 0.087). The younger group appear to eat less often while walking stairs, but also this difference is not significant (*p*= 0.32). Gender differences in the three behaviors (looking, *p*= 0.91, talking, *p*= 0.49, and eating, *p*= 0.70) were smaller and not significant either. Similar results were obtained when splitting the data for past falls, where people with no experience of falls tended to report less eating (*p*= 0.076).

## Discussion

The present study extended earlier work on visual attention during stair walking and the influence of concurrent tasks (Miyasike-daSilva et al. 2011; Miyasike-daSilva and McIlroy 2012, 2016; Lester et al. 2016; Zietz and Hollands 2009) to a real world scenario, in which participants walked stairs with or without using their phone. The results showed that phone use reduced dwell times on the stairs and surrounding areas to around 20% of the time, but did little to the distribution of attention within these 20% compared to when no phone was used. Walking time was increased by phone use, but walking time in itself did not influence the distribution of attention. Handrail use was low overall, was slightly reduced during phone use, but unaffected by age or previous experience with falls on stairs. The majority of participants reported to engage in concurrent tasks while walking stairs, including looking at one’s phone, talking on the phone and eating, with no differences across age groups, gender, or past experience with falls. Together, these results suggest that phones strongly distract attention away from the stairs and surrounding areas, which was compensated for by walking more slowly, but not by increased rail use.

The results raise the question of how participants manage to safely walk the stairs with very little time spent looking at them, particularly because past studies have shown that impaired vision makes locomotion and navigation more difficult and results in more falls (Buckley et al. 2005; Hassan et al. 2007; Hayhoe et al. 2009; Johnson et al. 2007; Lord et al. 2002; Marigold and Patla 2008; Timmis et al. 2009). A possible explanation is that people rely on memory either from earlier encounters of the stairs, or from inspection of the stairs before starting their ascent or descent (Rosenbaum 2009). Such an explanation, however, is at odds with findings by Miyasike-daSilva and McIlroy (2012), who found that preview of the stairs and repeated ascent and descent did not influence gaze behavior on the stairs. As a possible clue, we noticed that when considering views away from the phone, the distribution of attention was remarkably similar to when no phone was used, in particular concerning fixations on the stairs (when not using the phone, people looked more at the wall, which may be explained by the posters displayed). Furthermore, participants slowed down their walking when using their phone. Together these two strategies may have allowed people to safely walk the stairs without running into other people or tripping over the steps.

As in previous studies (Cohen and Cohen 2001; Miyasike-daSilva et al. 2011; Miyasike-daSilva and McIlroy 2012, 2016), the handrail was not often used, or visually inspected. We did, however, find more frequent inspection and handrail use than in previous studies. In our study, the handrail was fixated around 10% of the time and slightly less than half of the participants used the handrail. In contrast, Miyasike-daSilva et al. (2011) found that less than 5% of the time was spent looking at the handrail and only 3 out of 11 participants used the handrail, while Cohen and Cohen (2001) also found that around one third of the participants used the handrail. Possible reasons why the handrail was not often looked at may be that participants remembered its position from their first inspection of the stairs, that they could locate it in their peripheral vision (participants often looked near to, but not at the handrail), or that brief looks at the handrail suffice. There are also reasons why handrail use was higher in our study: Our stairs were visually more complex, were made of concrete, not wood or covered with carpet (making falls more risky), we had other people on the stairs, and our sample contained more elderly participants. Further studies are needed to establish exactly what determines handrail use, which could make use of both observation and of questionnaires probing the factors that people think influences their decision on using the handrail.

Interestingly, we found that people looked four or more steps ahead most of the times (for stair fixations), while more controlled stair walking studies found previews of between two to four (Miyasike-daSilva et al. 2011), or three steps ahead (Zietz and Hollands 2009). A possible reason for the further looking ahead could be that other stair users could be encountered on the stairs with whom collisions needed to be avoided (Jovancevic-Misic et al. 2007; Jovancevic-Misic and Hayhoe 2009), but also that our sample had a broader age range. Previous work has suggested that older adults more frequently look two steps ahead, while younger adults more often looked four steps ahead (Zietz and Hollands 2009).

In agreement with previous work, we found that participants walked the stairs more slowly when using the phone. Past work has shown that when walking in a field with obstacles, participants walked slower, and raised their leg higher when stepping over the obstacle while using a phone (Chen et al. 2016; Kim et al. 2015; Licence et al. 2015). For one our participants, phone use appeared to be particularly distracting. This participant adopted a strategy of alternating phone use and stepping to avoid having to engage in both activities simultaneously. In general, we noticed that participants varied in the ease with which they used their phone. Studies in other domains, such as surgery (for a review, see Hermens et al. 2013), chess playing (Reingold et al. 2001) and golf putting (Vine et al. 2011) has suggested that eye movements of experts and novices differ. Future studies could examine whether similar findings are obtained for mobile phone use in day-to-day activities, where expert phone users may be better at coordinating their phone use with activities such as stair walking and obstacle avoidance during normal walking.

For the majority of participants the stairs were familiar, particularly stairs 2 and 3. Previous work in a more controlled setting has shown no effect of previous encounters with stairs on gaze behavior (Miyasike-daSilva et al. 2011), which is consistent with the lack of clear differences in gaze behavior between the more familiar (stairs 2 and 3) and less familiar stairs (stairs 1) in the present study, although any of such differences may have been confounded by differences in properties of stairs.

Our results extend findings in locomotion (Hyman et al. 2010; Stavrinos et al. 2009; Schwebel et al. 2012) and driving (Konstantopoulos et al. 2010; Lamble et al. 1999; Strayer et al. 2003), showing that mobile phone use strongly attracts attention away from the current task and towards the phone. For locomotion, apps have been developed that use the phone’s camera to provide warnings to the pedestrian of possible risks during the walk (Foerster et al. 2014; Wang et al. 2012), which has been extended to cars (You et al. 2013). Alternative approaches are to use sensors in the phone to detect locomotion, and to warn the phone user of the dangers of using the phone while walking (Datta et al. 2014; Musić et al. 2013; Zhou 2015). On the basis of the present results, we anticipate that future developments of such technology towards detection of stair locomotion may be highly beneficial, with the aim of developing phone application that warn phone users when walking the stairs while engaged with their phone (e.g., texting, playing games) of the realistic dangers of their behavior.

The present study only looked at one possible risk factor for falls: Distraction from mobile phone use. A broad range of risk factors have been identified, including environmental (e.g., loose carpets, objects on stairs), medication (e.g., sedatives), medical conditions (e.g., Parkinson’s, rheumatic arthritis) or reduced vision (e.g., macular degeneration), nutritional (e.g, vitamin D deficiencies), and lack of exercise (Masud and Morris 2001). It is therefore important to study the role of these other risk factors and eye tracking may provide a valuable tool. For example, it may detect whether people see the objects on the stairs, how people compensate for vision problems, and how medications influence stair walking. Eye tracking may also benefit the design of stairways to determine how the layout of the stairs (Pauls 1991) and handrails (Maki et al. 1998) may aid in fall prevention.

### Conclusions

The present work extends earlier studies on visual attention during stair locomotion to a real world situation. The results show that mobile phone use strongly attracts attention away from the stairs, but that when gazing away from the phone, people distribute their attention similarly to when no phone is used. Handrail use is low, but slightly higher than in studies in more controlled settings, which could be due to the visual complexity of real world stairs, or the presence of other people on the stairs. The findings could be used to inspire mobile phone app developers to include the detection of stair locomotion in their apps, and to warn walkers not to use their phone while walking the stairs.
